# Stimulation of GLUT4 Glucose Uptake by Anthocyanin-Rich Extract from Black Rice (*Oryza sativa* L.) via PI3K/Akt and AMPK/p38 MAPK Signaling in C2C12 Cells

**DOI:** 10.3390/metabo12090856

**Published:** 2022-09-12

**Authors:** Shui-Yuan Feng, Shu-Jing Wu, Yun-Ching Chang, Lean-Teik Ng, Sue-Joan Chang

**Affiliations:** 1 Department of Life Sciences, National Cheng Kung University, Tainan 70101, Taiwan; 2 Department of Nutritional Health, Chia-Nan University of Pharmacy and Science, Tainan 71710, Taiwan; 3 School of Medicine, College of Medicine, I-Shou University, Kaohsiung 82445, Taiwan; 4 Department of Agricultural Chemistry, National Taiwan University, Taipei 10617, Taiwan

**Keywords:** anthocyanin, black rice (*Oryza sativa* L.), glucose uptake, GLUT4

## Abstract

Anthocyanin from black rice was reported to have beneficial effects on diabetes, but the molecular mechanisms are still largely unknown. Black rice cultivated from different regions in Taiwan (Hualien and Changhua) were included in this study. Concentrations of anthocyanin were significantly higher using the ethanol extraction method than those using water; therefore, ethanol extracts from Hualien and Changhua black rice (HBRE and CBRE) were used for further investigation. 2-NBDG glucose uptake analysis revealed that both HBRE and CBRE promote glucose uptake in C2C12 myotubes. The membrane expression levels of GLUT4 and phosphorylation of IRS-1 also had been markedly increased by both HBRE and CBRE, which was in accordance with the glucose uptake results. CBRE did not affect the downstream of IRS-1 but significantly enhanced protein levels of p-AMPK/AMPK. In contrast, HBRE was shown to target various signaling participated in GLUT4 glucose uptake, including PI3K/Akt and the p38 MAPK/ERK. Overall, we demonstrated that anthocyanin-rich extracts from black rice stimulate GLUT4 glucose uptake via upregulation of PI3K/Akt and AMPK/p38 MAPK signaling in C2C12 myotubes. Our findings revealed that anthocyanin-rich black rice might be a promising functional food for the prevention and treatment of insulin resistance and diabetic hyperglycemia.

## 1. Introduction

According to the World Health Organization, 537 million adults are living with diabetes around the world in 2021, and this number is predicted to rise to 643 million by 2030 [1]. It is generally accepted that type 2 diabetes, the most common type of diabetes, results from the failure of proper glucose utilization. Skeletal muscle is responsible for the plurality of glucose disposal and is the major site of insulin resistance in patients with type 2 diabetes [2]. Normal glucose uptake and metabolism in skeletal muscles are essential to maintain glucose homeostasis. Glucose uptake into skeletal muscle requires trafficking of vesicles containing glucose transporter-4 (GLUT4) from intracellular storage compartments to the plasma membrane, which is initiated via insulin signaling cascade and exercise-induced contraction in skeletal muscle [3]. Skeletal muscle-specific GLUT4 knockout mice exhibit severe insulin resistance and glucose intolerance at an early age [4]. Overexpression of GLUT4 in the muscles of genetically diabetic mice (db/db) alleviates insulin resistance by raising both basal- and insulin-stimulated glucose transport [5]. Therefore, GLUT4 has been thought of as a therapeutic target for pharmacological intervention strategies to control diabetic hyperglycemia.

Initiation of the classic insulin signaling pathway in skeletal muscle begins with insulin binding to an insulin receptor (IR) and triggers tyrosine-phosphorylation of insulin receptor substrate 1 (IRS-1) [6]. Downstream of IRS-1, the pathway branches into at least two major signaling transduction routes [3,7]. In one pathway, the phosphorylated IRS recruits phosphatidylinositol 3 kinase (PI3K) via Src-homology-2 (SH2) domain on the p85 subunit. Subsequently, activation of the PI3K/AKT signaling leads to GLUT4 translocation and promotes the utilization of glucose [6,7]. The second pathway leads from Ras to mitogen-activated kinases (MAPK) being involved in the regulation of intrinsic activity of glucose transporters [3,7]. Moreover, cumulative evidence indicates that the activation of AMP-activated protein kinase (AMPK) through physiological stimulation and pharmacological activators leads to a significant increase of glucose uptake mediated by the translocation of GLUT4 [8,9].

Rice (*Oryza sativa* L.), the main staple food for more than 50% of the world’s population, provides carbohydrates and protein to the average daily diet to supplement with essential micronutrients [10]. Recently, pigmented rice has received increasing attention due to its nutritional values and health benefits, which is in large conferred by its abundant content of phenolic compounds. Of the pigmented rice varieties, black rice (*Oryza sativa* L.) containing rich anthocyanins in the aleurone layer has been regarded as a healthy food and widely consumed since ancient times in China and other Eastern Asia countries [11]. In addition to beneficial properties, including anti-cancer, anti-inflammation, and improvement in hyperlipidemia, anthocyanin-rich extract from black rice was shown to ameliorate insulin resistance and hyperglycemia in a fructose-fed and streptozotocin-induced diabetic animal model [12,13,14]. 

Although anthocyanin-rich extract from black rice was reported to have a beneficial effect on diabetes, limited studies have been performed on the molecular mechanism of black rice anthocyanins in glucose disposal. As mentioned above, GLUT4 is a crucial step in delineating the mechanism of regulated glucose transport in skeletal muscle. To this end, we hypothesized whether anthocyanin-rich extract from black rice has an effect on GLUT 4 translocation in membrane fractions of muscle C2C12 cells and also on the other key kinases-associated signaling pathways.

## 2. Materials and Methods

### 2.1. Preparation of Black Rice Extracts 

Hualien black rice (HBR, non-glutinous black rice, *Oryza sativa* L., harvested in June of 2014) was supplied from the Fuli Farmers’ association at Hualien, Taiwan. Changhua black rice (CBR, Tainung Sen Glutinous 24, *Oryza sativa* L., harvested in May of 2014) was supplied from the Agricultural Research Institute, Chiayi Agricultural Experiment Branch, Taiwan. 

One hundred grams (100 g) of HBR and CBR powder were taken in the Erlenmeyer flask with 1 L RO water and boiled in a water bath for 1 h. The extract was filtered with filter paper (Advantec No. 1, Japan), and the residue was re-extracted under the same conditions twice. The filtrates obtained from three separate extractions were combined, concentrated, and lyophilized. The yields of HBR water extraction (HBRE) were 17.73%, while that of CBR water extraction (CBRE) was 12.10%. At the same time, one hundred grams (100 g) of HBR and CBR powder were soaked with 1 L of ethanol (95%) for 6 days. The extraction was performed in a thermostatic cell at 25 °C, protected from light, under constant stirring. After processing, the extract was filtered, and the filtrates obtained from three separate extractions were combined, concentrated, and lyophilized. The yields of HBR and CBR ethanol extractions (HBRE and CBRE) were 24.25% and 22.00%, respectively. All extracts were stored at −20 °C in polyethylene bottles, protected from light. 

### 2.2. Cell Culture and Differentiation

Mouse myoblast cells (C2C12) purchased from the Food Industry Research and Development Institute (FIRDI, Taiwan) were grown in 90% DMEM medium supplemented with 10% FBS, 100 units/mL penicillin, and 100 μg/mL streptomycin. They were maintained at 37 ℃ in a humidified atmosphere of 5% CO_2_. During the differentiation period, cells were incubated with DMEM containing 2% horse serum at confluence and replaced every 2 days for the next 4 days. All experiments were performed on differentiated C2C12 myotubes. The cultured mediums and reagents mention above were obtained from Thermo Fisher Scientific Inc. (Waltham, MA, USA).

### 2.3. Cell Viability Assay

The percentage of survival cells was measured by MTT colorimetric assay (Abcam, UK). In brief, cells were treated with black rice extracts at different concentrations (0, 10, 50, and 100 μg/mL) for 24 h. Cells were then harvested and washed with PBS before adding 50 µL of FBS-free medium and 50 µL of MTT solution. After 3 h of incubation at 37 °C, 150 µL of MTT solvent was added; the plate was wrapped in foil, and then shaken on an orbital shaker for 15 min. The optical density was measured at 590 nm with a microplate reader. 

### 2.4. Total Anthocyanin Content Analysis 

Each extract was mixed thoroughly with 0.025 M potassium chloride buffer. The absorbance of the mixture was then measured at 515 and 700 nm against distilled water blank. Similarly, the above extracts were dissolved with sodium acetate buffer (pH 4.5) and the absorbance of these solutions was measured at the same wavelengths. The total anthocyanin content was expressed as the mean (mg of cyaniding-3-glucoside (Cy-3-G) equivalents per 100 g sample dry weight ± SD for triplicate samples). 

### 2.5. Glucose Uptake Analysis 

The C2C12 myotubes in glucose-free DMEM were incubated for 24 h with insulin (50 nM), HBRE, CBRE (10, 50, or 100 μg/mL), and 2-NBDG (100 μM). After removal of the culture medium, the cells were washed 3 times with cold PBS. The fluorescence intensity of cellular 2-NBDG was measured at an excitation wavelength of 485 nm and emission wavelength of 535 nm.

### 2.6. Western Blot Analysis

Cell lysates were prepared by RIPA buffer containing protease inhibitor cocktail and phosphatase inhibitors (Thermo Fisher Scientific Inc., Waltham, MA, USA) and the protein concentrations of cell lysates were then measured using the BCA protein assay kit (Bio-Rad, Hercules, CA, USA). The Mem-PER™ Plus Membrane Protein Extraction Kit (Thermo Fisher Scientific Inc., Waltham, MA, USA) was used to prepare the plasma membrane proteins according to the manufacturer’s instructions. Equal amounts of lysate protein were subjected to 10% SDS-PAGE and then transferred to a PVDF membrane (Immobilon-P; Merck Millipore, Burlington, MA, USA). After this, it was blocked with 5% (*w*/*v*) nonfat milk powder in a Tris-buffer that contains 0.05% (*v*/*v*) Tween-20 (TBST) at room temperature, and the membrane was incubated overnight at 4 °C with the primary antibodies (Table S1). The signal was detected by using a horseradish peroxidase-conjugated secondary antibody (Bio-Rad) and revealed with the enhanced chemiluminescence system from Pierce.

### 2.7. Statistical Analysis

Data are from three independent experiments performed in duplicate and expressed as means ± standard deviation (SD). The statistical analyses were carried out using Student’s *t*-test and one-way analysis of variance (ANOVA) with Tukey’s post hoc test. *p* < 0.05 was considered statistically significant.

## 3. Results

### 3.1. Effects of Black Rice Extracts on Cell Viability in C2C12 Myotubes

**Figure 1:**
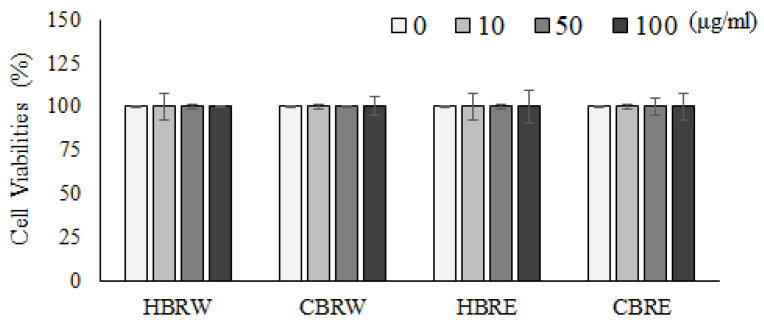
Cell viability of C2C12 myotubes in the presence of 0, 10, 50, and 100 μg/mL black rice extracts for 24 h. Data are presented means ± SD.

Hualien black rice (HBR) is cultivated and processed in the eastern region of Taiwan, and Changhua black rice (CBR) is cultivated and processed in the western region of Taiwan. Cytotoxic effects of black rice extract via ethanol and water extraction methods on cell viability were determined by MTT assay. After 24 h treatment of C2C12 myotubes with various concentrations of black rice extracts (10–100 μg/mL), the cell viability was not significantly affected (Figure 1). Concentration of anthocyanin, the active compound in black rice, was significantly higher in HBRE (7839 ± 117 mg/100 g) and CBRE (7678 ± 55 mg/100 g) than that in HBRW (242 ± 12 mg/100 g) and CBRW (34 ± 3 mg/100 g). Based on these data, HBRE and CBRE were further used to investigate the efficacy on the glucose uptake and underlying mechanism.

### 3.2. Effects of HBRE and CBRE on Glucose Uptake in C2C12 Myotubes

**Figure 2:**
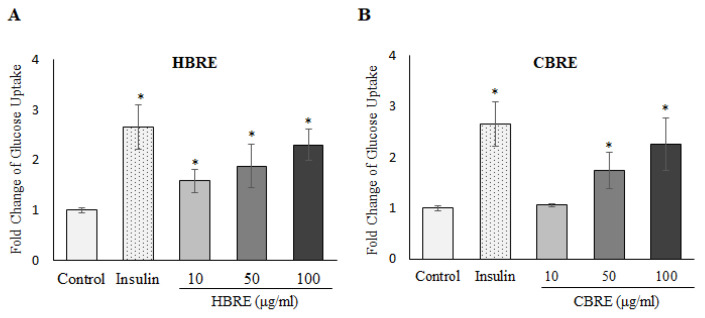
Effect of black rice extracts on glucose uptake by C2C12 myotubes. The 2-NBDG glucose uptake analysis was measured after a 24-h treatment with the indicated concentrations of (**A**) HBRE and (**B**) CBRE. Data are presented means ± SD, * *p* < 0.05 vs. control group.

**Figure 3:**
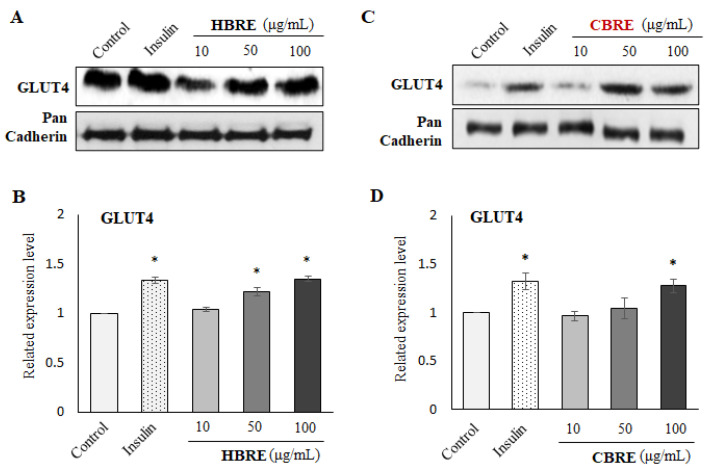
Effect of black rice extracts on GLUT4 protein expression in plasma membrane of C2C12 myotubes. Cells were treated with indicated concentrations of (**A**,**B**) HBRE and (**C**,**D**) CBRE for 24 h, and the representative immunoblot and quantification graphs were presented (means ± SD). * *p* < 0.05 vs. control group.

To evaluate the effect of black rice extracts treatment on glucose uptake, C2C12 myotubes were treated with 2-NBDG, a fluorescently labeled glucose analogue, with or without HBRE or CBRE, for 24 h. Results showed that glucose uptake was 2.66-fold higher for cells treated with 150 nM insulin. HBRE significantly increased glucose uptake in a dose-dependent manner from 10 to100 μg/mL (Figure 2A). CBRE significantly increased glucose uptake at higher concentrations, 50 and 100 μg/mL (Figure 2B). The marked increases in glucose uptake stimulated by both HBRE and CBRE at 100 μg/mL were even close to the level of the insulin-treated positive control. Both HBRE (Figure 3A,B) and CBRE (Figure 3C,D) significantly increased the expression of the membrane GLUT4 protein in C2C12 myotubes. Our data demonstrated that anthocyanin-rich black rice extracts stimulated glucose uptake mediated by GLUT4.

### 3.3. Effects of HBRE and CBRE on IRS/PI3K/Akt Signaling Pathways 

**Figure 4:**
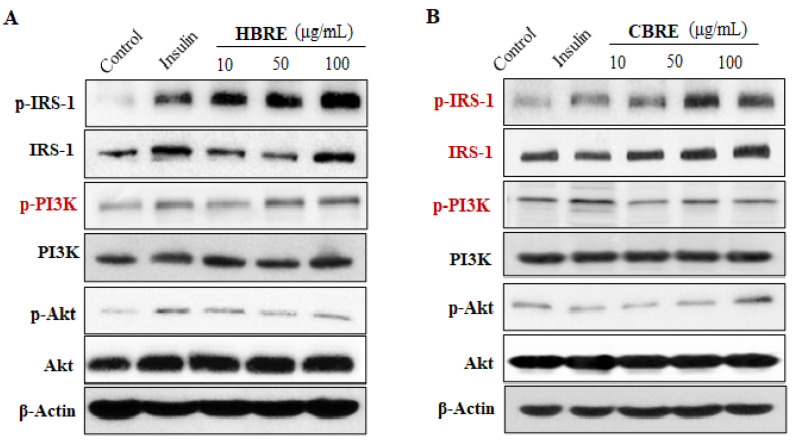

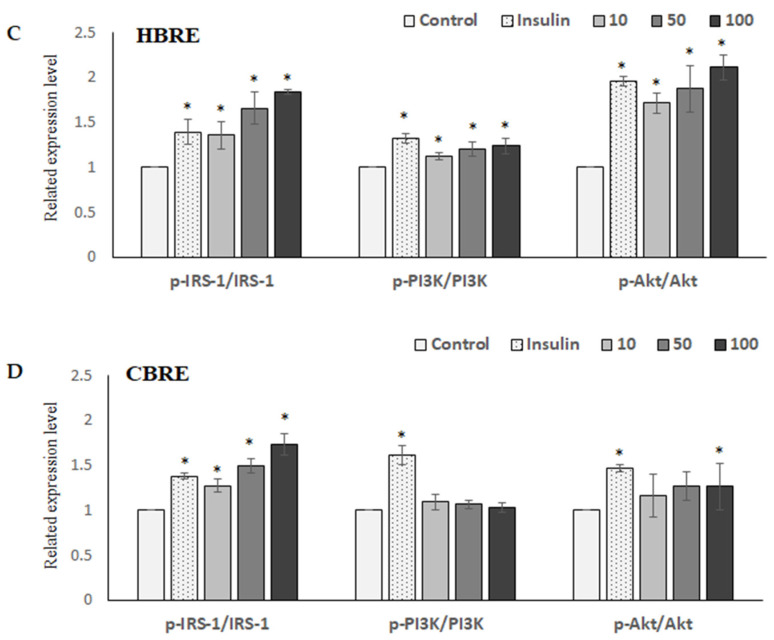
Effect of black rice extracts on protein expression of IRS/PI3K/Akt signaling in C2C12 myotubes. Cells were treated with indicated concentrations of (**A**,**C**) HBRE and (**B**,**D**) CBRE for 24 h, and the representative immunoblot and quantification graphs were presented (means ± SD). * *p* < 0.05 vs. control group.

Subsequently, we investigated the molecular mechanism of black rice extracts on the stimulation of GLUT4 expression in the plasma membranes of C2C12 myotubes. Results in Figure 4 revealed that both HBRE and CBRE significantly increased the ratio of p-IRS-1 to IRS-1 in C2C12 myotubes. It is noteworthy that only HBRE enhanced the phosphorylation of P13K and Akt (Figure 4A,C). These data suggested that HBRE stimulated glucose uptake via an IRS/PI3K/Akt signaling pathway; consequently, promoting the translocation of GLUT4 to the membrane. However, CBRE did not affect the signaling transduction of the P13K-Akt axis (Figure 4B,D), suggesting CBRE increased GLUT4-mediated glucose uptake through another pathway. 

### 3.4. Effects of HBRE and CBRE on AMPK/p38 MAPK/ERK Signaling Pathways

**Figure 5:**
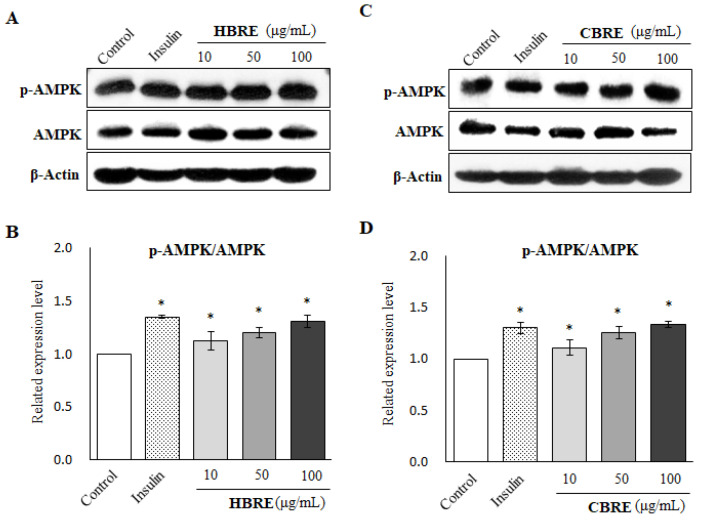
Effect of black rice extracts on AMPK signaling in C2C12 myotubes. Cells were treated with indicated concentrations of (**A**,**B**) HBRE and (**C**,**D**) CBRE for 24 h, and the representative immunoblot and quantification graphs were presented (means ± SD). * *p* < 0.05 vs. control group.

**Figure 6:**
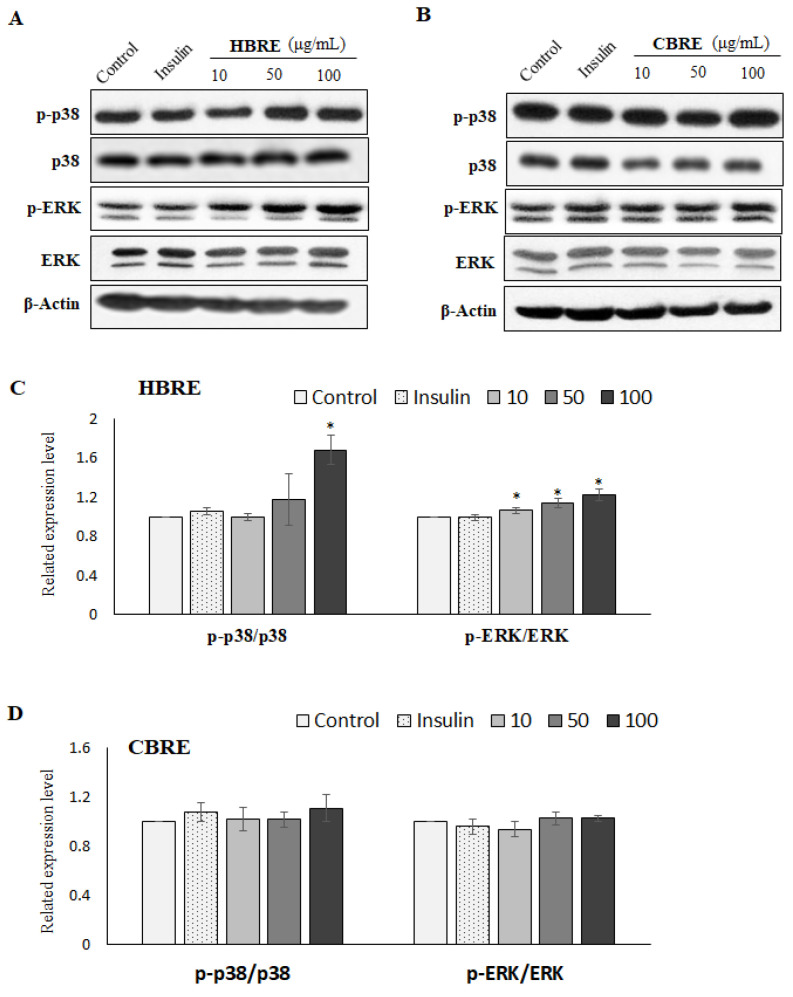
Effect of black rice extracts on protein expression of p38/ERK signaling in C2C12 myotubes. Cells were treated with indicated concentrations of (**A**,**C**) HBRE and (**B**,**D**) CBRE for 24 h, and the representative immunoblot and quantification graphs were presented (means ± SD). * *p* < 0.05 vs. control group.

Several studies support that AMPK can activate the downstream multiple kinases including p38 mitogen-activated protein kinases (p38 MAPK) and extracellular signal-regulated kinase (ERK), promote GLUT4 trafficking to the cell surface, and enhance glucose uptake in myotubes [8,15]. As we assume, both HBRE and CBRE significantly enhanced the ratio of p-AMPK (ser25) to AMPK (Figure 5A,B) in C2C12 myotubes. However, only HBRE significantly increased the phosphorylation of p38 MAPK and ERK (Figure 6A,C). CBRE did not affect the activity of both p38 MAPK and ERK in C2C12 myotubes (Figure 6B,D). Together, our results suggest that translocation of membrane GLUT4 stimulated by HBRE and CBRE may attribute to different pathways.

## 4. Discussion

Diabetes causes severe complications and disabilities that impose health and financial burdens on society. In addition to anti-hyperglycemic agents and insulin analogs, prevention and management strategies should also focus on lifestyle modification, such as changing exercise and eating habits [16]. Black rice, also known as purple rice or forbidden rice, exhibits the highest antioxidant activities of all the varieties of rice [11,17]. Its low glycemic index score of 42.3 and high fiber content (three times more than white rice) make it a healthier choice for those with high blood sugar, diabetes, or insulin resistance [18]. In Taiwan, the cultivation and consumption of black rice is becoming popular with local farmers and people with health concerns. The present study was performed with the aim of preparing black rice extracts collected from eastern (Hualien) and western (Changhua) regions, analysis of its phytochemical content, and evaluation of underlying molecular mechanisms of black rice extracts in glucose uptake by an in vitro cell model.

Phytochemical profiles of black rice are characterized by the presence of anthocyanin, which is one of the potential candidates to prevent and treat diabetes via protecting beta-cells, improving insulin resistance, increasing insulin secretion, and inhibiting carbohydrate hydrolyzing enzymes, as well as their antioxidant capacity [19]. 

The anthocyanin content of black rice is mainly determined not only by the genotype-variety [20], but is also affected by different growth environments. The first step in isolating anthocyanins from black rice is extraction, and the extraction efficiency is influenced by the solvent used. As our results show, concentrations of anthocyanin were significantly higher using the ethanol extraction method than those using water. Concentrations of anthocyanin of black rice from the eastern (Hualien) region were slightly higher than those from the western (Changhua) region.

The glucose transporter GLUT4 is critical for skeletal muscle glucose uptake and maintains whole body glucose homeostasis. Many reports showed that anthocyanin compounds, such as cyanidin-3-O-glucoside (Cy3G) and cyanidin3-rutinoside (C3R), could upregulate glucose uptake and GLUT4 expression in skeletal muscle cells and adipocytes [21,22]. In the current study, the rate of 2-NBDG uptake of HBRE and CBRE treatment groups was significantly increased compared with the control group (Figure 2). The membrane expression level of GLUT4 was also markedly increased (Figure 3), which was in accordance with the glucose uptake results. These findings suggested that anthocyanin-rich extracts from black rice could increase glucose uptake in C2C12 myotubes through the promotion of GLUT4 expression in the plasma membrane. Interestingly, HBRE seems more efficient in the stimulation of GLUT4 glucose uptake since it acts at lower concentrations. 

Insulin-stimulated trafficking of GLUT4 storage vesicles is initiated via two main branches: the PI3K-Akt and the Ras-MAPK pathways [3]. In the PI3K/Akt/GLUT4 axis, PI3K-p85 phosphorylate, its downstream protein Akt, and the p-Akt will then promote the translocation of GLUT4 to the cell surface, which, in turn, enhances the glucose uptake [3]. Anthocyanins from black soybeans promote glucose uptake in L6 rat skeletal muscle cells via upregulating phosphorylated Akt and GLUT4 [21]. The anthocyanin derivative C3R upregulates glucose uptake and PM-GLUT4 expression in 3T3-L1 adipocytes by activating the PI3K/Akt pathway [22]. The activated IR, like many other tyrosine kinases, induces the formation of Ras-GTP and the activation of MAPK. GTP-binding proteins are involved in the translocation of GLUT4-containing vesicles to the plasma, suggesting the Ras/MAP kinase pathway acts in the signaling pathway of insulin toward glucose uptake [23,24]. However, most studies on effects of Ras-MAPK signaling pathways by anthocyanins were carried out in cancer research [25,26]. In the present study, we found that both HBRE and CBRE stimulated the phosphorylation of IRS-1 in a dose-dependent manner from 10 to 100 μg/mL. Only HBRE significantly increased the protein levels of P13K, p-Akt/Akt, p-p38/p38, and p-ERK/ERK in C2C12 myotubes (Figure 4 and Figure 6), which showed that HBRE activated both PI3K/Akt and the Ras/MAPK/ERK signaling pathways. 

AMPK is another important mediator of glucose homeostasis via insulin-dependent/insulin-independent mechanisms. In the insulin-independent pathway, pharmacological and physiological stimuli activate AMPK, which stimulates glucose uptake by promoting GLUT4 translocation into the plasma membrane [27,28]. AMPK activators, such as AICAR and A-769662, promote GLUT4 trafficking to the cell surface and enhance glucose uptake in myotubes [9,29]. It has been demonstrated that anthocyanin-rich bilberry extract ameliorates hyperglycemia and insulin sensitivity via the activation of AMP-activated protein kinase in diabetic mice [30]. Consistently, our data revealed that anthocyanin-rich extracts from both HBR and CBR significantly enhanced protein levels of p-AMPK/AMPK in C2C12 myotubes (Figure 5A).

The differences of genetic background and cultivated environments between HBR and CBR may contribute varied phytochemical compounds and bioactivities. Therefore, GLUT4 glucose uptake stimulated by HBRE and CBRE may attribute to different pathways. HBRE seems more efficient in the stimulation of GLUT4 glucose uptake and this may result from the potential to target multiple signaling pathways. In addition to GLUT4, the evidence suggests that Akt and p38 MAPK signaling pathways are involved in regulating the expression of Glut1 proteins, which can promote glucose uptake and metabolism [31,32]. Further investigation is needed to determine whether black rice stimulates glucose uptake via Glut1 and the underlying signaling.

## 5. Conclusions

To conclude, our results support the hypothesis that the anthocyanin-rich extract from black rice promotes glucose uptake by increasing GLUT4 expression in the plasma membrane. We showed that the anthocyanin-rich extract from black rice increases glucose uptake in C2C12 myotubes via activation of the PI3K/Akt and AMPK/p38 MAPK pathways. Our results provide useful information which add value to black rice, supporting its use as an ingredient in functional food for the prevention and treatment of insulin resistance and diabetic hyperglycemia.

## Data Availability

Data for this study are available upon request from the corresponding author.

## Supplementary Materials

The following supporting information can be downloaded at: https://www.mdpi.com/article/10.3390/metabo12090856/s1, Table S1: List of antibodies used in the studies.
